# Use of a modified World Café process to discuss and set priorities for a Community of Practice supporting implementation of ReQoL a new mental health and quality of life Patient Reported Outcome Measure (PROM)

**DOI:** 10.1186/s41687-020-00202-z

**Published:** 2020-05-19

**Authors:** Elizabeth Taylor Buck, Christine M. Smith, Amanda Lane, Anju Devianee Keetharuth, Tracey Young, Jo Cooke

**Affiliations:** 1 School of Health and Related Research (ScHARR), The University of Sheffield, Regent Court, 30 Regent St, Sheffield, S1 4DA UK; 2 School of Human and Health Sciences, Centre for Applied Research in Health, University of Huddersfield, Huddersfield, UK; 3 Health Sciences School, The University of Sheffield, Sheffield, UK

## Background

Patient reported outcome measures (PROMs) are a means of assessing the quality and effectiveness of care from the patient’s perspective [1]. However, the routine use of PROMs in clinical practice can be difficult to implement [2, 3]. New challenges arise at different stages of the implementation process and organisations need to invest time and financial resources into designing an appropriate strategy, information systems, providing technical support and preparing staff [4, 5].

Recovering Quality of Life (ReQoL) is a PROM that was specifically designed to measure mental health service users’ perspectives of recovery and quality of life [6–8]. It is a co-produced, service user-centred outcome measure tested by over 6000 mental health service users. It is able to detect change across a broad spectrum of mild to severe mental health conditions.

In May 2016 a licence to use the ReQoL measures became freely available to the NHS and publicly funded research. At the time of publication, 149 licences had been issued worldwide and eleven official translations had been made available. The team that developed ReQoL has continued to collaborate with mental health trusts across the country to support the implementation of ReQoL. The National Institute for Health Research and Applied Research Collaboration (NIHR ARC YH^1^), and the preceding NIHR CLAHRC-YH, along with the ReQoL development team have helped to guide and fund this work. The NIHR CLAHRC-YH supported two national events, the first of which was the launch of ReQoL at the Houses of Parliament in October 2016. More recently in November 2018, over 70 people, from 23 organisations, attended a second event focused on the development of a ReQoL Community of Practice.

Community of Practices (CoPs) have existed in sectors such as education and business for over 30 years [9], operating as networks and support groups for people who share a common set of problems or interests. Members of CoPs maintain and build links with each other for the purposes of social interaction, knowledge sharing, knowledge creation and identity building [9, 10].

## World Café process

A collaborative interactive workshop took place during a national event aimed at supporting the implementation of ReQoL in practice. Invitations to the event were sent to people who had contacted the ReQoL team for support with implementation or expressed an interest following the launch of ReQoL two years earlier. An electronic ticketing website was used to publicise the free event and to allocate tickets. We sought to include as many different parties from as many different organisations as possible. All delegates attending the event were invited to participate in the World Café workshop.

**Fig. 1:**
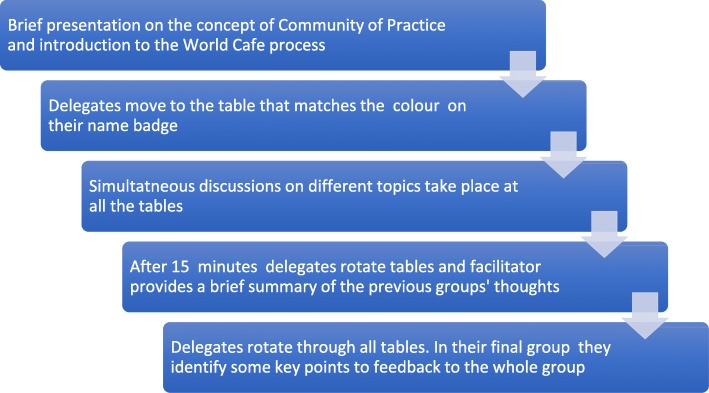
The modified World Café Process

A modified ‘World Café’ process [11] was run to explore delegates’ expectations and priorities for a ReQoL CoP. The aim of this short report is to describe and reflect on this process. The World Café process aims to bring people together to discuss issues that matter to them, tapping into tacit wisdom through interactive group discussions [11, 12]. The process allows participants to engage in evolving rounds of dialogue in smaller groups while remaining connected to a larger conversation [11]. The process can last several hours, however, in this case, the process was modified to fit into a 60-min workshop (see Fig. 1). Similar modifications have been used at other events [12, 13].

The theme of the workshop was ‘Establishing a Community of Practice for ReQoL: being creative about next steps together’. At the start of the workshop a brief presentation was given outlining the benefits of working within communities of practice. An overview of the World Café process was also provided so participants would understand the process. Participants were asked to present themselves at a table that matched a colour sticker on their name badge that had been randomly allocated by the conference organisers. At each table, a facilitator hosted conversations focussed on four previously agreed topics:

- Connecting people: How do you want to interact?
- Maintaining dialogue: How will you overcome barriers?
- Setting priorities: What will you work on?
- Impact: How will you know if you are making a difference?

Participants had 10 min to discuss the topics at each table. Their thoughts were captured by facilitators (JC, ETB, CS and TY) using post-it notes or flip-charts. Once participants had rotated around all the tables, the facilitators from each topic provided feedback on key points to the whole group. The structure of the workshop is summarised in Fig. 1.

## Data collection and analysis

The data was recorded and collected in the form of notes, lists and key phrases, on flip-chart sheets and post-it notes. After the workshop, two of the facilitators (JC and ETB) transcribed these notes, producing an initial account of the discussions. Thematic analysis of the initial account was undertaken by ETB. This involved: familiarisation with the data; coding; searching for themes; reviewing, defining and naming themes [14]. The themes were discussed and agreed upon with the other facilitators and a summary was sent to participants for comment.

## Results

A total of 71 people attended the whole event representing mental health trusts, independent organisations, academic institutions and government agencies (see Table 1). The workshop took place at the end of the event with an estimated 48 participants.

**Table 1:** Breakdown of people attending the event by organisation and job role

Organisation by type	Number attending event	Job roles represented
NHS Trusts	45	Consultant in Clinical and Medical Psychology
		Clinical Outcome Lead
		Psychiatric Nurse
		Clinical Director
		Psychotherapist
		Clinical Psychologists
		Support Worker
		Health Information Analyst Specialist
Academic Institutions	12	Professor of Economics
		Professor of Health Related Research
		Business Manager
		Research Fellow
		Health Economist
		Professor of Clinical Psychology
		Health Service Researcher
Independent organisations	7	Clinical Director
		Regional Manager
		National Health Sub Committee Member
		Staff Nurse
Government Agencies	7	Programme Director
		Mental Health and Learning Disability Commissioner
		Senior Project Manager
		Clinical Director

Analysis of the data collected during the workshop identified five key themes which were: Making links; Sharing learning and tasks; Creating change. Each theme will be explored separately below.

### Making links

Participants suggested that members of the CoP should meet regularly. Suggestions for face to face meetings included quarterly meetings with alternating locations, an annual one-day national meeting and more regular local meetings. The use of video conferencing software such as Skype and Zoom was also suggested to overcome geographical barriers. It was also suggested that webinars could be hosted on applications such as WhatsApp or Pebble Pad. It was agreed that a degree of passion would be needed, with everyone taking an active a role in making and maintaining links.

The idea of an on-line discussion forum on the ReQoL website was also put forward. It was proposed that there could be specific threads relating to different issues. Participants highlighted that the forum would need to be password protected, be compliant with data protection regulations, and might need a moderator or co-ordinator. It was suggested that the ReQoL team could take this role on, however it was acknowledged that this would require funding. Potential sources of funding or support were suggested including: commercial licence fees; charities; NHS England infrastructure team. It was also suggested that it might be worth joining forces with other networks or groups that had similar aims.

A buddy system was also proposed, so people in different organisations could link-up and support each other. Buddies could be identified by physical proximity; however, geographical location was not seen to be as important as finding people who are at the same stage of implementation.

### Sharing learning and tasks

One of the key priorities delegates identified for the CoP was to share learning about ReQoL implementation. There was particular interest in sharing learning about how to get formal, and informal “top down” and “bottom up” support for implementation. Delegates also wanted to learn from each other about: co-producing ReQoL implementation strategies with service users; launching ReQoL in different settings; embedding ReQoL in teams; building reports that enhance clinical usefulness; engaging service users with ReQoL so they feel they own their data; sharing service user stories; communicating the value of PROMs to staff; using ReQoL to develop outcomes based care-plans; tracking post-service outcomes including the trajectory from secondary to primary care; evaluating the use of ReQoL in services.

A CoP was also seen as having the potential to prevent duplication of tasks. People thought that by sharing tasks across organisations, progress could be made more quickly. One of the key priorities highlighted was developing an electronic service user facing “app” that could connect to different EPRs. It was noted that one of the challenges to sharing tasks would be the significant differences in organisational structures and processes, not least the raft of different EPRs used by NHS mental health trusts. However, technical and governance issues were raised around the app, in terms of where it would be hosted and the implications around safeguard of patients’ records.

The CoP was perceived as a forum in which people could collaboratively build knowledge and develop research, for example: building knowledge about the validity of ReQoL with different groups; identifying factors in the delivery of services and interventions that affect the outcomes that are achieved; teasing out different [clinical] approaches for different conditions, teams or services.

### Creating change

A key priority, identified for the CoP, was the use of ReQoL to patients by illustrating benefits of particular interventions or practices as evidenced by ReQoL with a view to influencing mental health policy, funders and commissioners, in both the NHS and the third sector. ReQoL was seen as having the potential to identify areas where increased (or adapted) service provision was required and to be a way of evidencing these unmet needs.

ReQoL was recognised as having the potential to support service development: changing attitudes in workforce; putting patient’s views at the centre of care; listening to clients; and focusing on recovery. ReQoL was seen as helpful for: collaborative planning; evaluating care plans; helping to get services connected with each other; putting client data in the centre; demonstrating where services are making a difference; supporting supervision and training; and setting service delivery targets with a realistic method of data collection.

In addition, participants emphasised the potential for ReQoL to support conversations between service users and staff: promoting ownership of care planning and outcomes, demonstrating progress, and supporting self-care.

Participants thought that they could support each other to create these changes and capture the impact of what they have done. They could also support their teams to think about engaging in research and service development, using outcomes to improve the quality of research and therefore evidence based practice.

## Discussion

The collaborative workshop demonstrated that there is an appetite for a ReQoL CoP amongst key stakeholders in organisations implementing, or planning to implement ReQoL. Participants identified priorities for the CoP which were: making links; sharing learning and tasks and creating change. They expressed a wish to maintain and develop links with each other via an on-line discussion forum and a programme of local and national events.

The modified World Café process offered participants an opportunity to influence the way the CoP was set up, commenting on what would work best for them and ensuring it is more likely to succeed. The process proved to be a quick and efficient way to elicit information, albeit requiring skilled facilitators and high levels of engagement from participants particularly when high numbers of participants are involved. On reflection we found it to be a useful technique for time-pressured co-production.

It is clear from the results that participants thought that developing an infrastructure to support the CoP would be key. Organisations seeking to establish similar CoPs would need to consider how the necessary infrastructure can be funded, something which could present a challenge in the NHS and social care at the current time.

### Next steps

Following the workshop, the ReQoL team has collaborated with a number of members of the CoP to set up an online discussion forum. Content for this has been created by CoP members and covers learning topics that were identified as important to share within the workshop. Additionally, a further face-to-face CoP event is being hosted in October 2019 to explore further some of the priorities identified.

## Conclusion

We have provided an example where developers of a measure are actively involved in the implementation of the measure. Our modified World Café process workshop confirmed an appetite for a ReQoL CoP which would enable people working in different organisations to facilitate implementation.

## Data Availability

The datasets analysed during the current study are available from the corresponding author on reasonable request.

## Abbreviations

CoP: Community of Practice
EPR: Electronic Patient Record
NHS: National Health Service
NIHR: National Institute for Health Research
PROM: Patient Reported Outcome Measure
ReQoL: Recovering Quality of Life
UK: United Kingdom

## Footnotes

1 ARCs are large research-practice collaborations funded by the National Institute for Health Research (NIHR) See: https://www.arc-yh.nihr.ac.uk/home
